# Editorial

**Published:** 1912-12

**Authors:** 


### EDITORIAL

The Eusiness Office of the *Journal-Record* is 23 West Alabama Street.

The Editorial Office is 1014-15 Atlanta National Bank Building.

Address all Business Communications to *Journal-Record of Medicine*, 23 West Alabama Street.

Make remittances payable to *The Journal-Record of Medicine*.

On matters pertaining to the Editorial and Original Communications, address Edgar G. Ballenger, M. D., Atlanta, Ga.

Reprints of Original Articles will be furnished at cost price. Requests for the same should always be made in THE MANUSCRIPT.

We will present, postpaid, on request to each contributor of an original article, twenty (20) marked copies of *The Journal-Record of Medicine* containing such article.

### MEDICAL SOCIOLOGY

We are now passing through a notable perIod in medicine. Not only have we made wonderful advances in recent years but the entire management of diseased individuals is undergoing a change. In Great Brittain this has reached a critical period. The Brittish Medical Association has severed negotations with the Government in regard to the Lloyd George bill and has obtained the resignations of nearly all physicians, holding contract practices with friendly societies and similar health insurance companies. Over 6,000 physicians have thus far sent in their resignations to take effect the first of the year unless the Government meets their demands. The only alternative the Government offers to have is the organization of a national medical service with full time physicians on salary. This of course the Government can do. Their national service for the Insurance Act would require 5,500 medical men and would cost about $15,000,000 annually. What the outcome will be remains to be seen. There is much food for thought in the entire situation. Will it soon reach us? New York, Boston, Chicago, and other cities are preparing men and women for the social service departments in hospitals and dispensaries. The increasing demand for persons so trained has lead the Social Service Department of the Boston Dispensary and of the Massachusetts General Hospital to offer the following practical course:

A. Introduction to the Medcial Institution Where Work is done.
 1. Classification of clinics.
 2. Admitting and distributing of patients.
  (a) Medical record system.
 3. Relation of institution to social service department.

B. Social Service Department.
 1. Organization.
 2. Function.
  (a) Relation to medical staff.
  (b) Relation to outside social agencies.
  (c) Relation to community.
 3. Technique of medical social worker in dispensary.
  (a) Personal approach to—
   (aa) Patients.
   (bb) Doctors.
   (cc) Institution o cers.
   (dd) Cooperating agencies.
  (b) Social records.
  (c) Registration thru a central bureau.
  (d) Filing system.
  (e) Statistics.
  (f) Weekly conferences on problems arising in case work.
 4. Subjects for special field work.
  (a) Children's clinic.
  (b) Tuberculosis.
  (c) Diseases of the eye.
  (d) Work with syyphilitics.
  (e) Work with nervous patients.
  (f) Work with six problems.
  (g) Work with the handicapped.

In addition the following lecture conferences are offered:

A. Study of Function and Technique of Hospital Social Service through Case Teaching Fifteen Exercises.

B. Lecture Conferences concerning
 1. Diseases having social significance

(a) Fatigue.

(b) Tuberculosis.

(c) Some mental and nervous diseases.

(d) The mental element in disease.

(e) Orthopedic conditions.

(f) Crippling and incapacitating.

(h) Alcoholism and drug habits.

(i) Sphyilis.

(j) Gonorrhoea.

(k) Children's diseases.

(l) Hygiene of pregnancy.

(m) Accidents and emergencies.

2. Eugencies.

3. Opportunities for research in the material of the day's work.

4. Governmental mechanism for the control of disease.

(a) Federal.

(b) State.

(c) Municipal.

5. Vital statistics.

6. Public health movements.

(a) The anti-tuberculosis campaign.

(b) Housing.

(c) Prevention of blindness.

(d) Prevention of infant mortality.

(e) Pure milk supply.

C. The Ethics and Ideals of the Medical Profession.

1. The interrelation of medical and social work.

D. The Development of Visiting Nursing.

E. Medical Social Servic. Its Development, Organization, and Future.

The medicine of the future is to become more and more prophylactic. Individual treatment will make way for socialistic and preventive medicine.

